# Supplementary material for: Serum-Derived Exosomal MicroRNA Profiles Can Predict Poor Survival Outcomes in Patients with Extranodal Natural Killer/T-Cell Lymphoma
Source: Cancers (Basel). 2020 Nov 27;12(12):3548. doi: 10.3390/cancers12123548 (PMC7761501; doi:10.3390/cancers12123548)

The unedited gel blots used in the Figures. The cropped Bands (in the blue boxes) are reported as Figure 1C.

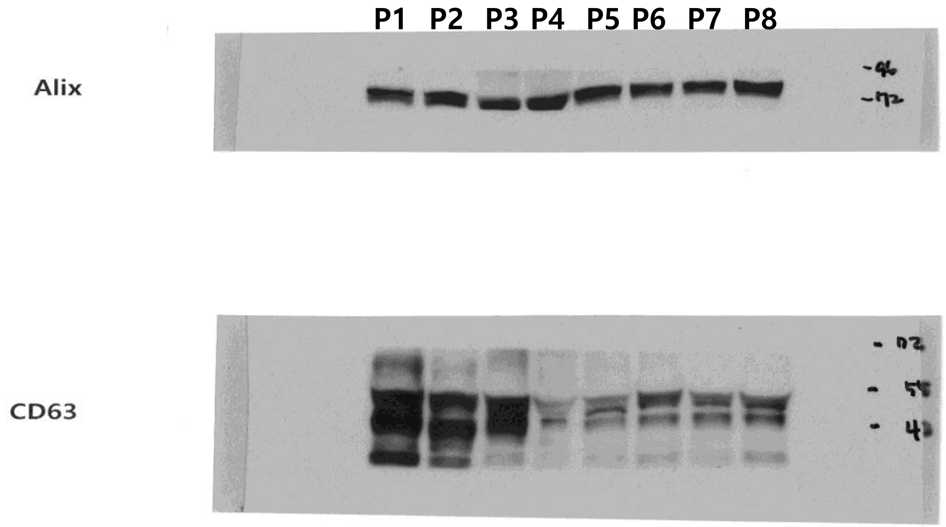

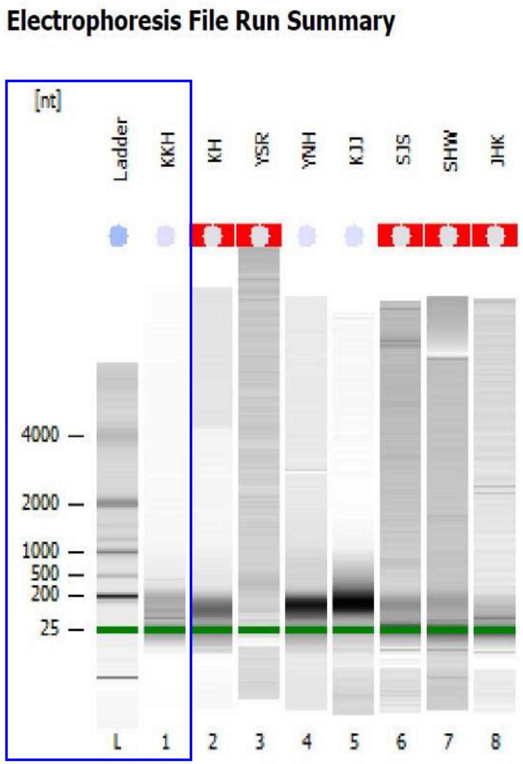

The unedited gel blots used in the Figures. The cropped Bands are reported as Figure 4.

Figure 4D

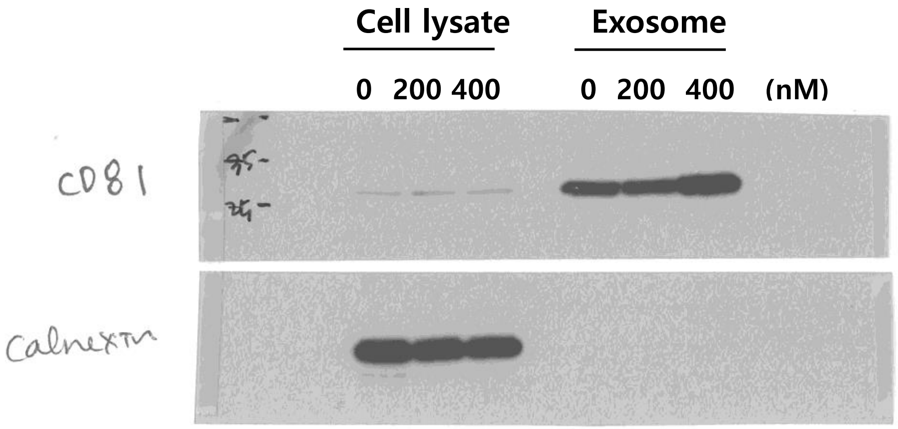

Figure 4G

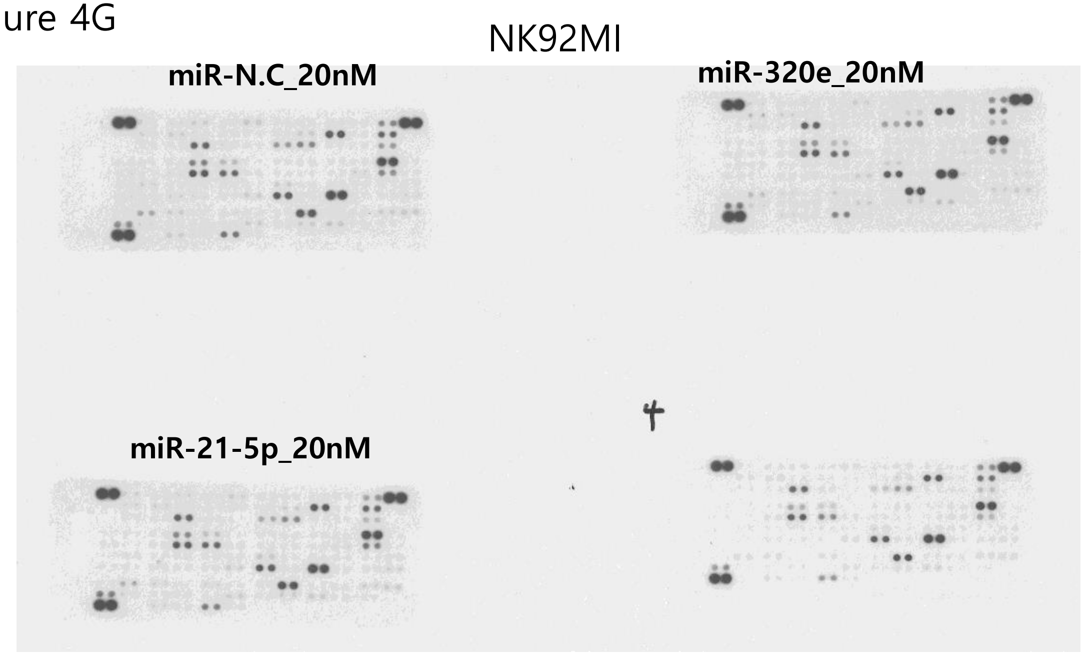

Figure 4H

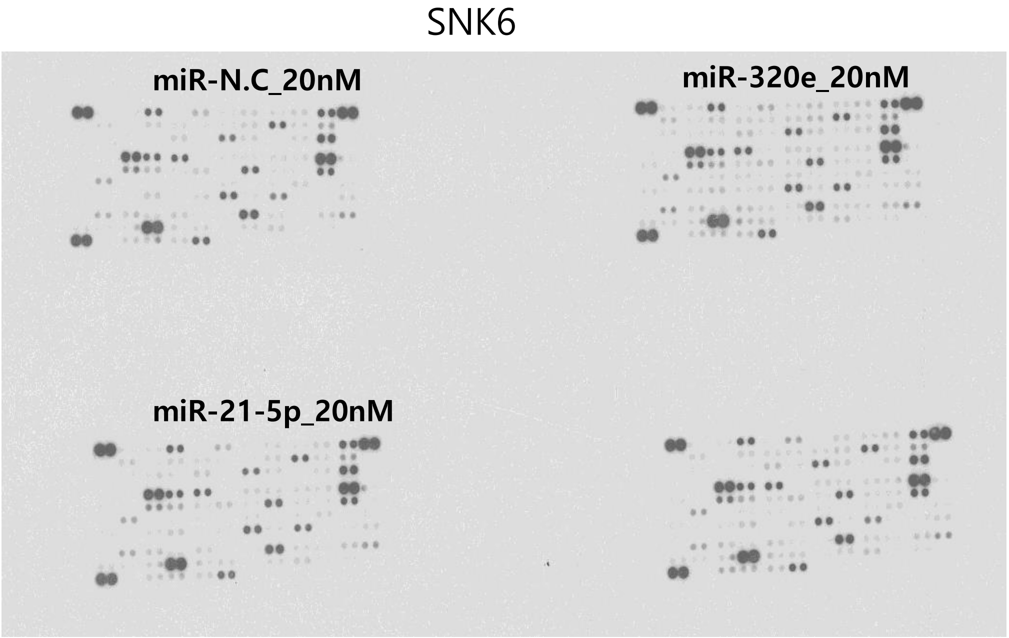

Supplement: Supplementary file 1 [file cancers-12-03548-s001.pdf]
